# Supplementary material for: Neurodevelopmental Outcomes at 18 Months of Corrected Age for Late Preterm Infants Born at 34 and 35 Gestational Weeks
Source: Int J Environ Res Public Health. 2021 Jan 13;18(2):640. doi: 10.3390/ijerph18020640 (PMC7828522; doi:10.3390/ijerph18020640)
Supplement: Supplementary file 1 [file ijerph-18-00640-s001.pdf]

Supplementary Table 1. Clinical characteristics of the late preterm infants born at 34 gestational weeks with and without follow-up data.

	LPI-34 with f/u data<br>*n* = 62	LPI-34 without f/u data<br>*n* = 27	*p* value
**Maternal data**			
Maternal age, year	34 (18–42)	35 (21-49)	0.42
Threatened preterm labor	36 (58)	17 (63)	0.67
Premature rupture of membrane	16 (26)	7 (26)	0.99
Hypertensive disorder of pregnancy	13 (21)	3 (11)	0.27
Gestational diabetes	9 (15)	3 (11)	0.67
Smoking	0 (0)	1 (4)	0.13
Multiple pregnancy	24 (39)	6 (22)	0.13
Cesarean section	53 (85)	21 (78)	0.37
Outborn	3 (5)	1 (4)	0.81
**Neonatal data**			
Male	39 (63)	14 (52)	0.33
BW, g	2061 (1354–2798)	2184 (1622-2930)	0.08
Apgar score at 1 min	8 (3–10)	8 (3-8)	0.87
Apgar score at 5 min	9 (6–10)	9 (6-9)	0.36
Small for gestational age infants	14 (23)	3 (11)	0.21
Artificial mechanical ventilation	44 (71)	20 (74)	0.76
Respiratory distress syndrome	17 (27)	10 (37)	0.36
Hyperbilirubinemia	47 (78) *	23 (85)	0.46
Severe hyperbilirubinemia	4 (7) #	4 (15)	0.22
Hypoglycemia	40 (67) *	14 (52)	0.19
Intraventricular hemorrhage	2 (3)	0 (0)	0.35
Periventricular leukomalacia	1 (2)	0 (0)	0.51
Length of hospital stay, day	29.5 (17–91)	23 (8-49)	0.03

Data are expressed as the medians (range), means ± standard deviations, or number (%).
All intraventricular hemorrhage were at grade Ⅰ.
f/u, follow up; BW, birthweight; DQ, developmental quotient; LPI-34, late preterm infants born at 34 gestational weeks.
* n=60 (two missing data)

Supplementary Table 2. Clinical characteristics of the late preterm infants born at 35 gestational weeks with and without follow-up data.

	LPI-35 with f/u data<br>*n* = 73	LPI-35 without f/u data<br>*n* = 41	*p* value
**Maternal data**			
Maternal age, year	35 (18–41) *	33 (23-42)	0.17
Threatened preterm labor	44 (60)	27 (66)	0.56
Premature rupture of membrane	25 (35)	12 (29)	0.59
Hypertensive disorder of pregnancy	10 (14)	3 (7)	0.30
Gestational diabetes	15 (21)	7 (17)	0.65
Smoking	2 (3)	4 (11) #	0.07
Multiple pregnancy	16 (22)	14 (34)	0.15
Cesarean section	45 (62)	27 (66)	0.65
Outborn	7 (10)	9 (22)	0.07
**Neonatal data**			
Male	42 (58)	17 (41)	0.10
BW, g	2328 (1700–3562)	2272 (1702-3392)	0.50
Apgar score at 1 min	8 (2–9)	8 (6-9)	0.32
Apgar score at 5 min	9 (4–10)	9 (8-10)	0.18
Small for gestational age infants	10 (14)	5 (12)	0.82
Artificial mechanical ventilation	33 (45)	16 (39)	0.52
Respiratory distress syndrome	6 (8)	3 (7)	0.86
Hyperbilirubinemia	40 (55)	25 (61)	0.52
Severe hyperbilirubinemia	8 (11)	2 (5)	0.27
Hypoglycemia	34 (47)	23 (58) $	0.27
Intraventricular hemorrhage	1 (1)	0 (0)	0.45
Periventricular leukomalacia	0 (0)	0 (0)	＞0.99
Length of hospital stay, day	19 (9–60)	20 (10-50)	0.08

Data are expressed as the medians (range), means ± standard deviations, or number (%).
All intraventricular hemorrhage were at grade Ⅰ.
f/u, follow up; BW, birthweight; DQ, developmental quotient; LPI-35, late preterm infants born at 35 gestational weeks.
* n=72 (one missing data)
# n=36（five missing data）
$ n=40 (one missing data)
